# The Influence of Environmental, Biotic and Spatial Factors on Diatom Metacommunity Structure in Swedish Headwater Streams

**DOI:** 10.1371/journal.pone.0072237

**Published:** 2013-08-15

**Authors:** Emma Göthe, David G. Angeler, Steffi Gottschalk, Stefan Löfgren, Leonard Sandin

**Affiliations:** 1 Department of Aquatic Sciences and Assessment, Swedish University of Agricultural Sciences, Uppsala, Sweden; 2 Department of Bioscience, Aarhus University, Silkeborg, Denmark; Consiglio Nazionale delle Ricerche (CNR), Italy

## Abstract

Stream assemblages are structured by a combination of local (environmental filtering and biotic interactions) and regional factors (e.g., dispersal related processes). The relative importance of environmental and spatial (i.e., regional) factors structuring stream assemblages has been frequently assessed in previous large-scale studies, but biotic predictors (potentially reflecting local biotic interactions) have rarely been included. Diatoms may be useful for studying the effect of trophic interactions on community structure since: (1) a majority of experimental studies shows significant grazing effects on diatom species composition, and (2) assemblages can be divided into guilds that have different susceptibility to grazing. We used a dataset from boreal headwater streams in south-central Sweden (covering a spatial extent of ∼14000 km^2^), which included information about diatom taxonomic composition, abundance of invertebrate grazers (biotic factor), environmental (physicochemical) and spatial factors (obtained through spatial eigenfunction analyses). We assessed the relative importance of environmental, biotic, and spatial factors structuring diatom assemblages, and performed separate analyses on different diatom guilds. Our results showed that the diatom assemblages were mainly structured by environmental factors. However, unique spatial and biological gradients, specific to different guilds and unrelated to each other, were also evident. We conclude that biological predictors, in combination with environmental and spatial variables, can reveal a more complete picture of the local vs. regional control of species assemblages in lotic environments. Biotic factors should therefore not be overlooked in applied research since they can capture additional local control and therefore increase accuracy and performance of predictive models. The inclusion of biotic predictors did, however, not significantly influence the unique fraction explained by spatial factors, which suggests low bias in previous assessments of unique regional control of stream assemblages.

## Introduction

One of the most frequently studied topics in ecology is the relationship between species distributions and local environmental factors [1]–[4]. For stream systems, it was early recognised that communities are affected by factors operating at different spatial scales [5], [6]. However, recently this knowledge has been reframed, incorporating metacommunity theory [7], [8], which emphasises that local communities are constrained by a combination of regional (e.g., landscape connectivity and dispersal related mechanisms) and local (environmental filtering and biological interactions) factors [9], [10]. Accordingly, four metacommunity concepts can be used to describe geographical patterns of biodiversity of streams [9], [10]. Species sorting and mass effects assume the importance of an environmental gradient and dispersal. Species sorting posits that dispersal is sufficient for species to be ‘sorted along’ an environmental gradient, whereas mass-effects become apparent when high dispersal, in addition to environmental factors, alters species composition [11], [12]. By contrast, neutrality and patch dynamics assume that patches are identical (i.e., no environmental gradient) and that dispersal is limited. The neutral concept emphasises the importance of stochastic events on community composition, including ecological drift, while the patch-dynamics concept assumes that empty patches are always available because dispersal rates are too low to compensate for local extinctions. Patch dynamics considers the relevance of biological interactions between and colonisation potential of organisms and invokes a competition-colonisation trade-off (i.e., strong dispersers are weak competitors and vice versa) [9], [10].

While the effect of biotic interactions and dispersal on species community structure has been strongly emphasised in metacommunity research, applied research has traditionally focused greatly on environmental control of freshwater assemblages, which is also reflected in current bioassessment and management approaches [8]. However, all four metacommunity paradigms are recognised as important for structuring stream communities [13]–[16], emphasising the importance of local (environmental filtering, biotic interactions) and regional factors. In previous studies, local environmental and spatial (regional) factors have been studied concurrently [15]–[19]. By contrast, few studies have included both environmental and biological variables as predictors of community composition (e.g., streams [20] and lakes [21]). Nevertheless, these few studies support the importance of species sorting along biological gradients (i.e., effects on community composition stemming from local species interactions), highlighting that the inclusion of biotic predictors may improve model accuracy. Moreover, recent studies have shown that dispersal-mediated trophic interactions (i.e., dispersal of species at one trophic level affects interactions with species at other trophic levels) can appear as unique spatial structures in species assemblages, thus overestimating the unique fraction explained by regional factors [22]. This suggests that biotic interactions are not only mediated by environmental factors [23]–[26] but also by spatial factors. To strengthen inferences, all three predictors (environmental, biotic, and spatial factors) should therefore be included in models [21], [22].

Here, we assess the relative importance of environmental, biotic and spatial factors using diatoms as our model organisms. Diatom assemblages are not only useful for assessing the relative role of environmental and spatial factors [17], [27]–[30], but also for assessing the relevance of biological species sorting. First, many small-scale experiments have been carried out to study the effect of grazing on diatom communities, with most showing significant effects of grazing on assemblage composition and biomass [31]. Second, as sensitivity to grazing pressure varies among guilds [32], this information can be used to assess the relative importance of grazing. We use a dataset from boreal headwater streams (first order stream reaches) in south-central Sweden (study area extent: ∼14000 km^2^), which included information about diatom taxonomic composition, invertebrate grazer abundances (biotic factor), environmental (physicochemical) and spatial factors (obtained through spatial eigenfunction analyses). The main aims of this paper were to investigate whether local biotic predictors can explain additional variation of taxonomic composition [20] and whether their inclusion significantly affects the detection of unique spatial structures [22]. That is, the likelihood of underestimating local control vs. overestimating regional control of stream assemblages. We strengthened inference by performing separate analyses on different diatom guilds with different sensitivities to grazing. More specifically, we predicted that different diatom guilds show (1) a similar, strong response to the environmental gradient, (2) a similar weak response to the spatial gradient due to the small study area (i.e. no dispersal limitation) and (3) different responses to the biotic gradient due to differences in species sensitivities to grazing.

## Materials and Methods

### Ethics Statement

The sampling was done in accordance with Swedish national and regional regulations and no permissions were necessary for the field work and collection. The field studies did not involve endangered or protected species.

### Study Area

**Figure 1:**
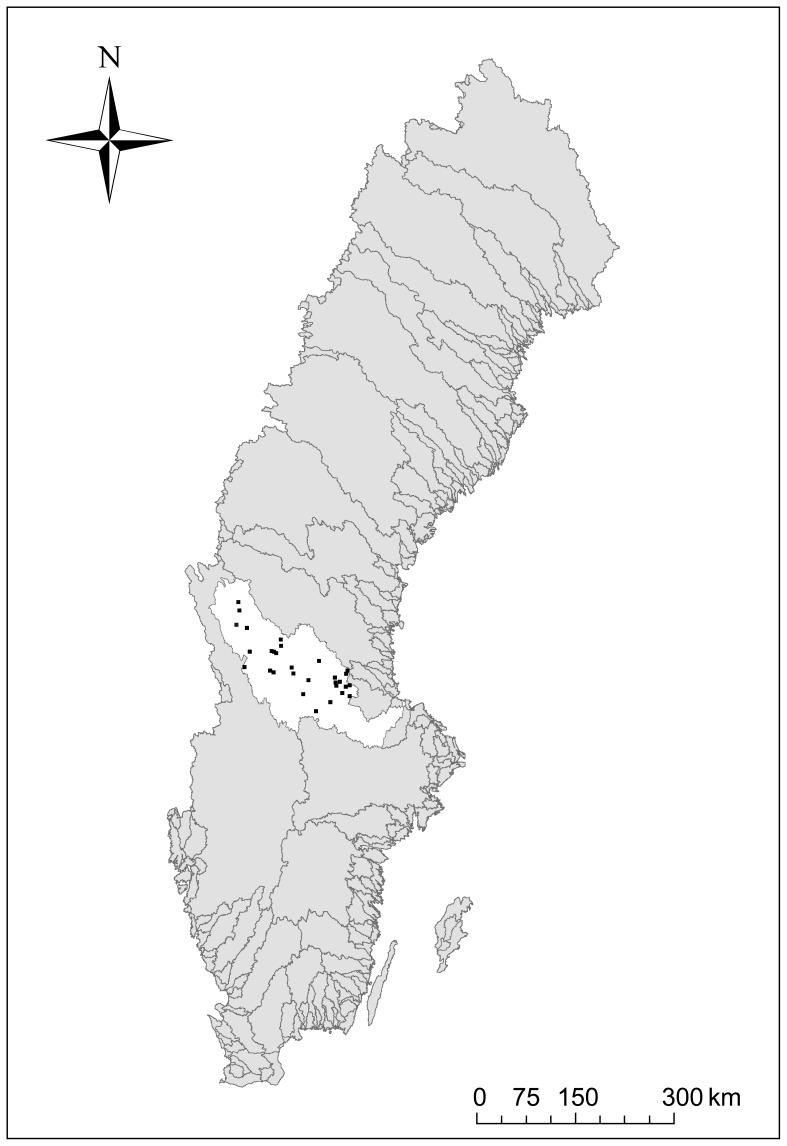
Location of the study area (Dalälven catchment) (white area) and the sampling sites (black dots) in Sweden.

The study area (ca 14000 km^2^) is located in the River Dalälven catchment (29000 km^2^) in south-central Sweden (Fig. 1). The study area varies markedly in underlying bedrock and soils, resulting in relatively strong gradients in water chemistry. In addition, the study sites cover an altitudinal gradient ranging 146–631 m a.s.l. (Table 1).

**Table 1:** Summary of environmental variables across the 30 study sites in the Dalälven River catchment.

Environmental variable	Mean	Min	Max	SE	Trans
Catchment area (ha)	198	106	363	11	log_10_
Altitude (m)	321	146	631	22	square-root
Depth (cm)	15	9	24	1	none
Width (m)	1.1	0.4	2.1	0.1	log_10_
Canopy cover (%)	62	32	78	2	none
Flow (m s^−1^)	0.23	0.11	0.44	0.01	square-root
Fine dead wood (no 50 m^−1^)	10	0	27	1	none
Coarse dead wood (no/50 m^−1^)	6	0	27	1	log_10_
Wetlands (%)	3.6	0	8.3	0.4	square-root
Clearcuts (%)	5.7	0	25.5	1.4	square-root
Lake (%)	0.4	0	5.5	0.2	square-root
pH	5.95	4.31	7.26	0.14	none
Conductivity (mS m^−1^)	2.58	1.61	4.29	0.11	log_10_
ANC (mekv l^−1^)	0.22	0.08	0.44	0.02	none
Alkalinity (mekv l^−1^)	0.05	0	0.31	0.02	none
Tot-N (ug l^−1^)	486	62	1139	45	none
Tot-P (ug l^−1^)	11	2	28	1	none
TOC (mg l^−1^)	22.9	2.6	58.3	2.2	none
Absorbance (at 420 nm)	0.45	0.04	1.12	0.05	log_10_

Mean (mean), minimum (min) and maximum (max) values, standard error (SE), and the transformation made prior to statistical analyses (trans) are shown.

### Selection of Sampling Sites

The sampling sites used in this study are a subset of study sites randomly selected for a larger project dealing primarily with nutrient losses from boreal areas [33]. Based on GIS analyses and altitude data from the Swedish Land Survey, a virtual hydrological network was created for the River Dalälven consisting of almost 70000 stream reaches, of which approximately 26000 were first order headwaters. A subset of approximately 400 first order headwaters fulfilled the criteria: (1)>2500 m in length (perennial flow), (2)<500 m from a road juncture, (3) no human point sources of contamination, and (4)<5% agricultural land in the catchment. From this subset, 100 headwaters were randomly selected. We then selected a subset of 30 similar riffle-sites (suitable for both macroinvertebrate and diatom sampling). At each riffle-site, a 50 m long representative stretch was selected for habitat characterisation and the sampling of macroinvertebrates, diatoms, and water chemistry.

### Habitat Characterisation and Water Chemistry Sampling

Habitat characterisation was performed along the 50 m stretch in October (2009). Stream width, depth, flow and canopy cover were measured at 10 evenly spaced transects. Depth and flow was measured at three points at each transect, while width and canopy cover was measured once at each transect. Canopy cover was assessed using digital pictures taken from the middle of the stream (at the stream surface) pointing upwards. The pictures were manipulated in computer software Image-Tools (Health Science Center, University of Texas, U.S.A.) so that black pixels represented the canopy and white pixels represented open areas. The percentage of black pixels was then calculated and used as an estimation of mean canopy cover. The number of items of coarse dead wood (diameter>10 cm) and fine dead wood (diameter<10 cm) along the 50 m stretch was also counted. Water samples were collected in August-September 2009. Samples were analysed at the SWEDAC accredited laboratory at the Department of Aquatic Sciences and Assessment (SLU) for conductivity, pH, alkalinity, total organic carbon (TOC), nutrients (Total-N, Total-P) and water colour (absorbance at 420 nm) according to Swedish standards [34]. Acid neutralising capacity (ANC) was also calculated. The assessment of catchment characteristics (i.e., percentage of wetlands, clearcuts and lakes) were based on maps acquired from Swedish Land Survey and the Swedish forestry agency and calculated in ArcGIS (ESRI, Redlands, California, USA).

### Benthic Invertebrate Sampling

Benthic invertebrates were sampled at each 50 m stream reach in October (2009) using a Surber sampler (14×14 cm). At each site, 15 subsamples were collected (covering ∼0.30 m^2^) and preserved in 70% ethanol. In the laboratory, the animals were sorted against a white background and individuals identified to the lowest taxonomic unit possible (usually genus or species). Invertebrates were divided into functional feeding groups (according to [35], [36]) and abundances of grazer/collector taxa (taxa that graze and/or collect particles from the stream-bed) were extracted. For taxa that were not found in the references above, we used an online database for freshwater organisms (www.freshwaterecology.info) to obtain scores describing their feeding mode preferences. These taxa were classified as grazers if they were scored ≥50% grazing. Taxa within the grazer-collector guild were combined into orders (Ephemeroptera, Trichoptera, Plectoptera and Coleoptera) before constructing the biotic predictor matrix. This grouping was done to reduce the risk of overestimating the effect of rare grazer/collector species, without having to exclude them from the analysis. We did not simplify the biotic predictor matrix further, since invertebrate species may respond differently to environmental and spatial factors [14], [15] and any further simplification may bias the analysis. A summary of grazer abundances within each order can be found in Table 2.

**Table 2:** Summary of grazer-collector density (number m^−2^) within each invertebrate order across the 30 study sites in the Dalälven River catchment.

Order	Mean	Min	Max	SE	Trans
Ephemeroptera	395	0	1803	99	hellinger
Plecoptera	214	0	755	41	hellinger
Coleoptera	44	0	177	17	hellinger
Trichoptera	6	0	75	3	hellinger

Mean (mean), minimum (min) and maximum (max) values, standard error (SE), and the transformation made prior to statistical analyses (trans) are shown.

### Diatom Sampling

Diatoms were sampled in October (2009) according to the European/Swedish standard method (SS-EN 13946) by scraping biofilm from the upper surface of five cobbles using a toothbrush and pooling the contents into one composite sample. The composite samples were stored in plastic containers (250 ml) and preserved in ethanol (70%). In the laboratory the samples were then treated with H_2_O_2_ and embedded in Naphrax®. Approximately four hundred diatom valves from each sample were identified to species (with very few exceptions) according to the European/Swedish standard method (SS-EN 14407). Counting about 400 valves captures approximately 85% of all taxa present in the sample if the sample contains a total of 50 taxa [37]. Diatoms were classified into functional groups reflecting growth form (low, high and motile) or size of single cells (small and large) (see Table S1). The low-growth guild includes species that grow in the boundary layer of the biofilm, close to the substrate. The high-growing guild includes species that can grow beyond the boundary layer of the biofilm. The motile guild includes species that can actively move relatively fast [32]. The small guild consist of species having a length of single cells ≤15 µm, and a volume of single cells ≤100 µm^3^, while the large guild consist of species having a length of single cells>15 µm, and a volume of single cells>100 µm^3^. A general description of diatom abundance and diversity across and within the guilds is given in Table 3.

**Table 3:** Diversity indices (taxon richness, Shannon index, Simpson index and Eveness index) and abundance of all diatom taxa combined and within each guild (high-growth, low-growth, motile, small-sized and large-sized) across the 30 study sites in the River Dalälven catchment.

	Taxon richness	Abundance	Shannon index	Simpson index	Evenness index
All taxa	33±2	421±2	2.22±0.09	0.79±0.02	0.31±0.01
High	19±1	294±18	1.99±0.07	0.77±0.02	0.41±0.02
Low	6±1	98±19	0.88±0.10	0.42±0.04	0.56±0.05
Motile	6±1	28±6	1.12±0.11	0.53±0.05	0.69±0.04
Small	26±2	376±9	2.05±0.09	0.76±0.02	0.32±0.01
Large	6±0	46±9	1.16±0.09	0.57±0.04	0.62±0.04

Means ± standard error (SE) are shown. The abundance of all taxa combined is ∼400 since approximately 400 individuals were identified in each sample (see methods, diatom sampling). Thus, the abundances within guilds are relative abundances (of the total number of identified individuals∼400).

### Statistical Analyses

All statistical analyses were performed in statistical software R [38]. First, we created the diatom abundance (species×site) matrices, which were Hellinger transformed prior to further analyses. This transformation makes species data that contains many zeros suitable for linear methods (e.g., redundancy analysis [RDA]) [39]. Second, we created environmental, spatial and biotic predictor matrices. The environmental matrix consisted of a set of environmental variables describing water chemistry, hydromorphology and catchment characteristics of each site (Table 1). All environmental variables were checked for normality and log or square root transformed where necessary. The biotic (order×site) matrix consisted of grazer abundances, which were Hellinger transformed prior to further analysis [39]. The spatial matrix consisted of spatial variables calculated through Principal Coordinates of Neighbour Matrices analyses (PCNM) [40], [41], based on Euclidian distances between each pair of sampling sites. These spatial variables were created by using the function *pcnm* in R package vegan [42]. The PCNM analysis creates a number of spatial variables representing small to large-scale spatial structures across a study area (here 18 PCNMs with positive eigenvalues were created for further analysis). The first PCNMs represent large-scale structures, and successive PCNMs represent subsequent smaller spatial structures. The PCNMs can then be used to predict spatial patterns in species distributions [40], [41]. We selected spatial, environmental, and biotic variables that were significantly (p<0.05) related to diatom assemblage structure with a forward selection procedure using the function *forward.sel* in R package *packfor* [43], which is based on RDA. The significant variables were then retained for further (variation partitioning) analyses.

To assess the relationship between local (environmental and biotic) and regional (spatial) predictors and the structure of diatom assemblages, we used partial redundancy analysis (pRDA) (variation partitioning analyses) by using the function *varpart* in R package vegan [42]. This function calculates how much of the variance in species community structure can be explained uniquely by each explanatory matrix as well as the shared variance explained by the explanatory matrices (we report only adjusted R^2^ values in the results). The significance of each testable fraction in the variation partitioning analyses was obtained by using function *rda* in R package vegan [42]. These analyses were performed separately and in the exact same way for the entire diatom assemblage and each diatom guild.

## Results

### Correlates of Diatom Taxonomic Composition

The physicochemical variables pH, altitude, shading, and total phosphorus and spatial variables, reflecting large (PCNM 1, 3) to medium (PCNM 9) scale patterns, were significantly related to diatom taxonomic composition in the River Dalälven catchment. Of the biotic variables, the abundance of grazing Ephemeropterans and Trichopterans correlated significantly with diatom taxonomic composition (Table 4).

**Table 4:** Results from the forward selection procedure, showing significant environmental, spatial, and biotic variables selected when all taxa were analysed together and when different diatom guilds (high-growth, low-growth, motile, small-sized, and large-sized) were analysed separately.

	Environmental variables	AdjR2cum/F/p-value	Spatial variables	AdjR2cum/F/p-value	Biotic variables	AdjR2cum/F/p-value
**All taxa**	pH	0.23/9.96/0.001	PCNM1	0.08/3.37/0.006	Ephemeroptera	0.19/7.75/0.001
	Altitude	0.31/3.80/0.001	PCNM9	0.13/2.62/0.011	Trichoptera	0.22/2.27/0.015
	Shading	0.32/1.63/0.047	PCNM3	0.18/2.61/0.009		
	Tot-P	0.34/1.61/0.047				
**High**	pH	0.22/9.10/0.001	PCNM1	0.08/3.36/0.003	Ephemeroptera	0.18/7.49/0.001
	Altitude	0.31/4.63/0.001	PCNM9	0.14/3.20/0.006	Trichoptera	0.21/2.00/0.038
	Shading	0.33/1.73/0.044	PCNM3	0.21/3.32/0.005		
			PCNM6	0.24/1.94/0.046		
**Low**	pH	0.13/5.35/0.001	PCNM1	0.08/3.43/0.009	Ephemeroptera	0.11/4.57/0.003
			PCNM16	0.13/2.62/0.021	Coleoptera	0.15/2.46/0.029
**Motile**	pH	0.08/3.60/0.002	PCNM2	0.05/2.43/0.013	Trichoptera	0.08/3.41/0.002
	Altitude	0.16/3.74/0.002			Ephemeroptera	0.14/2.96/0.006
**Small**	pH	0.25/10.83/0.001	PCNM1	0.08/3.48/0.006	Ephemeroptera	0.21/8.93/0.002
	Altitude	0.33/4.05/0.001	PCNM3	0.13/2.76/0.013	Trichoptera	0.25/2.31/0.020
	Shading	0.34/1.70/0.044	PCNM9	0.19/2.91/0.010		
			PCNM5	0.22/1.94/0.046		
**Large**	Altitude	0.09/4.00/0.001	PCNM2	0.05/2.37/0.008	Trichoptera	0.08/3.61/0.001
	Depth	0.13/2.15/0.012	PCNM9	0.08/2.01/0.038	Ephemeroptera	0.11/1.87/0.041
	pH	0.16/2.02/0.021				
	Tot-P	0.20/2.21/0.023				
	Absorbance	0.23/2.17/0.008				

**Figure 2:**
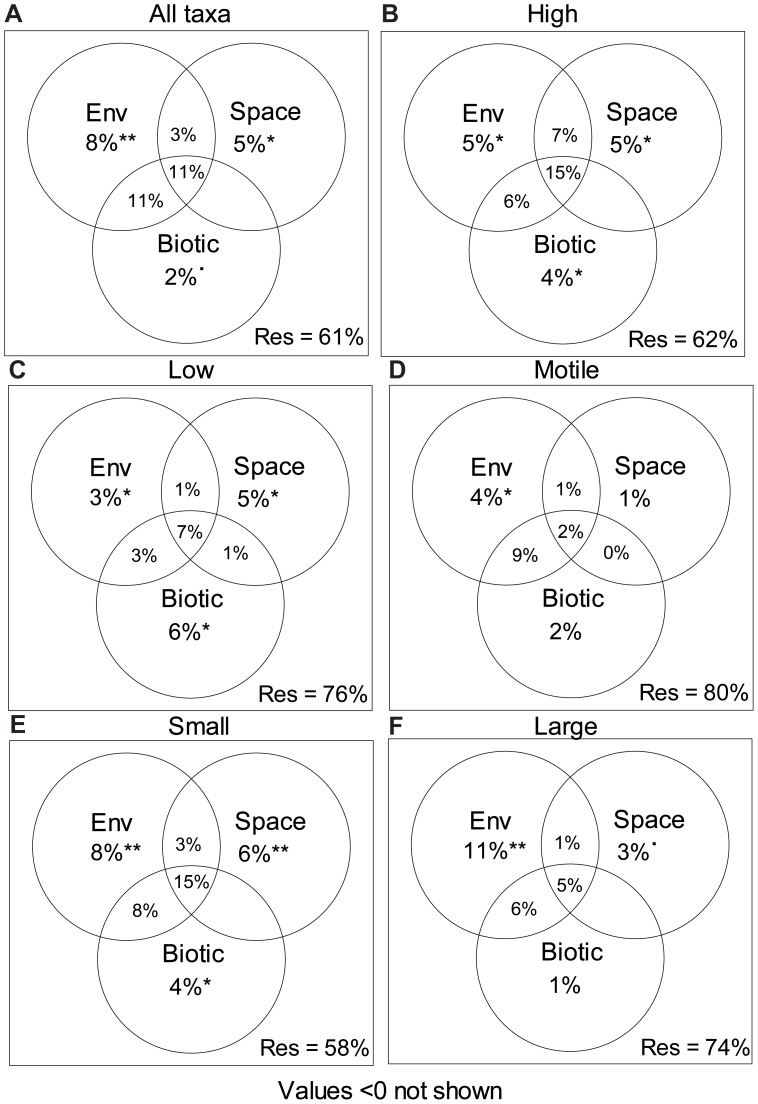
Venn-diagrams showing the unique fraction of taxonomic composition explained by environmental (Env), spatial (Space) and biotic (Biotic) factors. Figures show variances explained of a) total diatom assemblage structure (i.e., all taxa were analysed together) and of taxonomic composition within the b) high-growth, c) low-growth, d) motile, e) small-sized, and f) large-sized guild. The significance of each fraction explained is indicated in the figures (˙p<0.1, *p<0.05, **p<0.01). Shown is also the variance explained jointly (overlapping parts of the circles) by all three explanatory matrices, environmental and biotic factors, biotic and spatial factors, and environmental and spatial factors. The number of replicates (n) is 30 in all analyses. The residuals/unexplained variance (Res) in each analysis is reported under each figure, in the lower right hand corner. Note that the sum of all variances shown in the figure can exceed 100%. This is because variances can be negative [74], but these values are not shown (values<0 not shown).

Local environmental (8%) and spatial factors (5%) explained 13% of the diatom taxonomic composition, while the unique fraction explained by biotic factors (2%) was marginally significant (p<0.10). The variance explained jointly by environmental and spatial variables (spatially structured environmental variation) was 3%. Local biotic and environmental factors jointly explained 11% of taxonomic composition, with another 11% of the variance explained by the shared fraction between all three explanatory matrices. However, none of the explained variation was shared between spatial and biotic factors (Fig. 2a).

### Correlates of Diatom Taxonomic Composition within Guilds

Both pH and altitude were consistently related to the taxonomic composition of the diatom guilds. Other important environmental variables in one or more guilds were shading (high-growth and small-sized guild), depth, total phosphorus, and absorbance (large-sized guild). Spatial variables reflecting large-scale structures (PCNM 1, 2, 3) were consistently related to the taxonomic composition of all guilds. Medium scale structures (PCNM 5, 6, 9) were related to taxonomic composition of the high-growth, small-, and large-sized guilds, while small-scale structures (PCNM 16) were related only to the low-growth guild. Of the biotic factors, the abundance of grazing Ephemeropterans and Trichopterans was consistently related to the taxonomic composition of guilds, with the exception of Trichopterans and the low-growth guild. The abundance of grazing Coleopterans was related only to taxonomic composition of the low-growth guild (Table 4).

Local environmental factors were consistently and strongly related to the taxonomic composition of each diatom guild. Within each guild, environmental factors significantly explained 3–11% of the taxonomic composition and the shared variance explained jointly by environmental and spatial variables (spatially structured environmental variation) ranged from 1 to 7%. However, the unique amount of variation explained by spatial and biotic factors varied more between guilds. Spatial factors explained 5–6% of the variation in the high-growth, low-growth, and small-sized guild, but could not explain any unique fractions of taxonomic composition in the motile or large-sized guild. Similarly, biotic factors explained unique fractions of taxonomic composition within the high-growth, low-growth, and small-sized guild (4–6%), but not within the motile and large-sized guild. The shared explained variation between biotic and environmental factors ranged from 3 to 9% but basically no variation was shared between diatom spatial structures and biotic factors (0% or negative values within all guilds except the low-growth guild [1%]) (Fig. 2b–f).

## Discussion

Although observational studies like ours limit the assessment of mechanisms, the individual and collective patterns emerging from environmental, biological and spatial factors, and their shared variance, provided us with more detailed insight into what factors determine the structure of diatom assemblages in boreal headwater streams. The inclusion of the biological component was particularly relevant since it helped to increase our understanding of the potential role of biological interactions at the landscape scale in headwater streams, for which our current knowledge is still limited [44], [45].

Environmental and spatial factors explained unique and significant fractions of diatom taxonomic composition in the Dalälven catchment. Unique biological relationships between grazer abundances and diatom assemblage composition were only marginally significant when all diatom taxa were analysed together. The variance explained by environmental factors dominated over the spatial and biological fractions. This result supports the notion that stream ecosystems and their communities are under strong abiotic control [1], [3], [46]. pH varied substantially between the sites included in this study (range: 4.31–7.26) and was the variable that was most consistently related to diatom taxonomic composition. This finding highlights the sensitivity of diatoms to acid stress in aquatic ecosystems [47]. Invertebrate grazers that were used to construct the biological predictor matrix in this study are, similarly to the diatom assemblage, known to be sensitive to variation in acidity [48]. Redundancy analysis showed that the variation in the grazer guild was significantly associated with pH (AdjR^2^ = 0.22, p = 0.001) and the amount of shared variance between the environmental and biological fraction in the variation partitioning analyses also suggested that the grazer and diatom communities show a similar strong species sorting along acidity gradients in this catchment.

It was recently suggested that spatial structures in species data, originating from dispersal mediated trophic interactions, can appear as unique spatial structures in variation partitioning analyses, and therefore overestimate the fraction explained by regional factors [22]. In our study, however, the shared variance between spatial and biological predictors was almost nonexistent, suggesting that the inclusion of biotic predictors did not significantly influence the fraction explained by spatial factors. We suggest two non-mutually exclusive explanations for this finding. First, strong environmental effects may have overridden any potential spatial effects on trophic interactions between the grazers and the diatoms. For example, trophic interactions in freshwater habitats are often strongly mediated by environmental factors such as pH [25], [26], [49]–[51], which was the most pronounced gradient in our data set. Also, many structural features of stream food webs (web size, linkage density and complexity) may increase with pH [52]. Second, diatoms and invertebrates could be structured at different spatial scales due to differences in body size and dispersal traits [53]. Because dispersal capacity is expected to decrease with increasing body size [53], [54], invertebrates may be more dispersal limited compared to diatoms and consequently related to other spatial variables.

According to our predictions, biotic predictors explained unique and significant fractions of the variation in diatom assemblage composition, but only within specific guilds (i.e., the high-growth, low-growth, and small-size guilds). Experimental studies lend some support to our findings. For example, grazers have been shown to frequently ingest diatoms growing in the upper layer of the biofilm, decreasing their biomass [55], [56], while species growing closer to the substrate may increase in abundance [55], [57], probably due to release from competition. Also, small individuals have been shown to occur more frequently in grazer guts than large species [56] and motile diatoms are less selectively grazed compared to early succession non-motile and colonial forms [58]. In a previous study, the diversity of other taxonomic groups was a relatively poor predictor of diatom diversity (in comparison to invertebrate, macrophyte and fish diversity) [20]. Our study indicates that such correlations may be masked when analysing the entire diatom assemblage as a whole, and that an assessment of biological relationships can benefit from a trait or functional approach. Also, our study shows that the inclusion of biotic predictors can increase accuracy and performance of predictive models by detecting additional local control of lotic species assemblages.

In contrast to our predictions, spatial factors also had a significant effect on the structure of diatom assemblages despite the relatively small study area (∼14000 km^2^). The finding of pure spatial structures in diatom assemblage distribution is in line with previous studies covering both small [30], [59] and large spatial extents [17], [27], [29]. Studies over larger spatial extents have interpreted spatial structures to reflect dispersal limitation [27], [29], whereas studies covering smaller spatial extents seldom discussed alternative dispersal mechanisms [30], [59]. It could, however, be argued that any spatial structure observed over a small spatial extent (as in our study) is likely reflecting mass-effects due to relatively high connectivity (and thus high dispersal rates) among sites [9], [12], [60]. This is especially likely since diatoms and other small, unicellular organisms are thought to be distributed over very large spatial scales due to high dispersal rates [61]–[63]. This interpretation was also made by Astorga et al. [64] who found that diatoms were spatially structured at small (<200 km) but not at larger spatial extents, which implies mass-effects rather than dispersal limitation. Mass-effects could also occur through passive downstream movement within the stream channels [65], but since we did not have several sampling sites along each stream, we were not able to model downstream processes [66]–[68] and draw any conclusions about their importance for diatom distributions at this scale. However, irrespective of the causal dispersal mechanism behind pure spatial structures (i.e., low vs. high dispersal effects), our findings imply that it is not only important to maintain environmental conditions within local habitats, but also to maintain dispersal between habitats in the river landscape (e.g., identify source habitats and keeping dispersal pathways open) [69].

Interestingly, species within different guilds appeared to be differently structured along unique spatial gradients. Since we know very little about diatom dispersal (i.e., how they disperse, their dispersal capacities, and what traits determine their dispersal capacity) [70] we did not make any specific predictions regarding the spatial structuring of guilds. However, it has been suggested that locally abundant species are also globally abundant and therefore represent taxa that are more easily/frequently dispersed, while the opposite would be true for locally rare species [68], [71]. In our study, the large and motile diatom guilds (which were less spatially structured) were generally characterised by species with lower abundances in comparison with the small, high and low guilds (which were more spatially structured) (Table 3). The disparate findings between guilds could therefore reflect diatom traits related to dispersal capacity. We can only speculate what these traits consist of, but earlier studies have shown that size could potentially be an important factor. For example, a study of algae succession on Surtsey (a volcanic island south of Iceland) reported that the first colonisers were small, and that larger forms were absent within 3 years after the formation of the island [72]. In addition, another study found a relationship (although weak) between regional occupancy and diatom size [73].

Overall, our study shows that diatom assemblages in south-central Swedish headwater streams are mainly the result of local control. However, regional (spatial) gradients were also evident over this relatively small landscape scale, which may reflect high dispersal effects (mass effects). Most importantly, our study indicates that biological species sorting, which was seemingly unrelated to spatial gradients and guild specific, can be evident also at this scale of observation. Although biotic factors have largely been ignored in stream studies at similar or larger spatial scales, we show that it may be important to assess their effect on species assemblage structure, in addition to environmental and spatial predictors, or we risk underestimating the total local control of species communities. However, the inclusion of biotic predictors did not significantly alter the unique spatial fraction explained which implies low bias in previous assessment of local vs. regional control of stream assemblages.

## Supporting Information

Table S1 **List over the diatom taxa found in the 30 headwater sites investigated, indicating to which size (small/large) and growth-form guild (high/low/motile) each taxon was classified.** If information was not available for a taxa, this is indicated as n.c. = not classified.
(DOCX)
