# Supplementary material for: The Influence of Environmental, Biotic and Spatial Factors on Diatom Metacommunity Structure in Swedish Headwater Streams
Source: PLoS One. 2013 Aug 15;8(8):e72237. doi: 10.1371/journal.pone.0072237 (PMC3744466; doi:10.1371/journal.pone.0072237)
Supplement: Table S1 — List over the diatom taxa found in the 30 headwater sites investigated, indicating to which size (small/large) and growth-form guild (high/low/motile) each taxon was classified. If information was not available for a taxa, this is indicated as n.c. = not classified. (DOCX) [file pone.0072237.s001.docx]

| **Species** | **Author** | **Size** | **Growth-form** |
| --- | --- | --- | --- |
| Achnanthes species |  | small | low |
| Achnanthidium daonense | (Lange-Bertalot) Lange-Bertalot, Monnier & Ector | small | low |
| Achnanthidium helveticum | (Hustedt) Monnier, Lange-Bertalot & Ector | small | low |
| Achnanthidium kriegeri | (Krasske) Hamilton, Antoniades & Siver | small | low |
| Achnanthidium kranzii | Round & Bukhtiyarova | small | low |
| Achnanthidium subatomoides | (Hustedt) Monnier, Lange-Bertalot & Ector | small | low |
| Amphipleura kriegeriana | (Krasske) Hustedt | small | motile |
| Achnanthes linearioides | Lange-Bertalot | small | low |
| Achnanthidium minutissimum | (Kützing) Czarnecki | small | low |
| Amphora species |  | large | low |
| Aulacoseira alpigena | (Grunow) Krammer | large | high |
| Aulacoseira distans s.l. |  | large | high |
| Aulacoseira lacustris | (Grunow) Krammer | large | high |
| Aulacoseira species |  | large | high |
| Aulacoseira tenuistriata | Lange-Bertalot in ms | large | high |
| Brachysira brebissonii | Ross | large | motile |
| Brachysira neoexilis | Lange-Bertalot | large | motile |
| Brachysira serians | (Brébisson) Round & Mann | large | motile |
| Brachysira styriaca | (Grunow) Ross | large | motile |
| Caloneis tenuis | (Gregory) Krammer | large | motile |
| Cymbopleura naviculiformis | (Auerswald) Krammer | small | low |
| Chamaepinnularia mediocris | Lange-Bertalot | small | low |
| Chamaepinnularia soehrensis var. hassiaca | (Krasske) Lange-Bertalot | small | low |
| Chamaepinnularia soehrensis var. soehrensis | Lange-Bertalot & Krammer | small | low |
| Chamaepinnularia species |  | small | low |
| Cymbella naviculacea | Grunow | large | low |
| Cocconeis placentula incl. varieties | Ehrenberg | small | low |
| Diadesmis contenta var. contenta | Mann | small | n.c. |
| Diatoma mesodon | (Ehrenberg) Kützing | small | high |
| Diadesmis paracontenta | Lange-Bertalot & Werum | small | n.c. |
| Diadesmis perpusilla | (Grunow) D.G. Mann | small | n.c. |
| Diploneis peterseni | Hustedt | small | motile |
| Eunotia arcus | Ehrenberg | small | high |
| Eunotia arculus | (Grunow) Lange-Bertalot & Nörpel | small | high |
| Eunotia bilunaris var. bilunaris | (Ehrenberg) Mills | small | high |
| Eunotia bilunaris var. linearis | (Okuno) Lange-Bertalot & Nörpel | small | high |
| Eunotia bilunaris var. mucophila | Lange-Bertalot & Nörpel | small | high |
| Eunotia boreoalpina | Lange-Bertalot & Nörpel-Schempp | small | high |
| Eunotia botuliformis | Wild, Nörpel & Lange-Bertalot | small | high |
| Encyonopsis falaisensis | (Grunow) Krammer | small | low |
| Eunotia chelonia | Nörpel-Schempp | small | high |
| Eunotia circumborealis | Lange-Bertalot & Nörpel | small | high |
| Eunotia crista-galli | P.T.Cleve | small | high |
| Eunotia curtagrunowii | Nörpel-Schempp & Lange-Bertalot | small | high |
| Eunotia denticulata | (Brébisson) Rabenhorst | small | high |
| Encyonopsis descripta | (Hustedt) Krammer | small | low |
| Eunotia diodon | Ehrenberg | small | high |
| Eunotia exigua var. tenella | (Grunow) Nörpel & Alles | small | high |
| Eunotia exigua var. exigua | (Brébisson & Kützing) Rabenhorst | small | high |
| Eunotia exsecta | (Cleve-Euler) Nörpel-Schempp & Lange-Bertalot | small | high |
| Eunotia faba | Grunow | small | high |
| Eunotia flexuosa | (Brébisson) Kützing | small | high |
| Eunotia formica | Ehrenberg | small | high |
| Eunotia glacialis | Meister | small | high |
| Eunotia implicata | Nörpel, Lange-Bertalot & Alles | small | high |
| Eunotia incisa var. incisa | W. Smith & W. Gregory | small | high |
| Eunotia meisteri | Hustedt | small | high |
| Eunotia microcephala | Krasske | small | high |
| Eunotia minor | (Kützing) Grunow | small | high |
| Eunotia minor s.l. |  | small | high |
| Eunotia muscicola var. tridentula | Nörpel & Lange-Bertalot | small | high |
| Eunotia muscicola var. muscicola | Krasske | small | high |
| Encyonopsis species |  | small | low |
| Eunotia neocompacta | Mayama | small | high |
| Encyonema lunatum | (W. Smith in Greville) Van Heurck | small | low |
| Encyonema minutiforme | Krammer | small | low |
| Encyonema minutum | (Hilse) Mann | small | low |
| Encyonema neogracile var. neogracile | Krammer | small | low |
| Encyonema perpusillum | (A. Cleve) Mann | small | low |
| Eunotia nymanniana | Grunow | small | high |
| Eunotia pseudoparalleloides | (Grunow) Nörpel-Schempp & Lange-Bertalot | small | high |
| Eunotia pectinalis s.l. |  | small | high |
| Eunotia praerupta | Ehrenberg | small | high |
| Encyonema pergracile | Krammer | small | low |
| Eunotia paludosa var. trinacria | (Krasske) Nörpel | small | high |
| Eunotia rhomboidea | Hustedt | small | high |
| Eunotia septentrionalis | Østrup | small | high |
| Eunotia silvahercynia | Nörpel, Van Sull & Lange-Bertalot | small | high |
| Eunotia steineckei | Petersen | small | high |
| Eunotia subarcuatoides | Alles, Nörpel & Lange-Bertalot | small | high |
| Eunotia sudetica | O. Müller | small | high |
| Encyonopsis subminuta | Krammer & Reichardt | small | low |
| Eunotia tetraodon | Ehrenberg | small | high |
| Eunotia bidens | Ehrenberg | small | high |
| Eunotia groenlandica | (Grunow) Nörpel-Schempp & Lange-Bertalot | small | high |
| Eucocconeis laevis | Lange-Bertalot | small | low |
| Eunotia species |  | small | high |
| Eunotia paludosa var. paludosa | Grunow | small | high |
| Eunotia varioundulata | Nörpel & Lange-Bertalot | small | high |
| Fragilaria arcus var. arcus | (Ehrenberg) Cleve | small | high |
| Fragilaria capucina s.l. |  | small | high |
| Frustulia crassinervia | (Brébisson) Lange-Bertalot & Krammer | small | motile |
| Fragilaria capucina ssp. rumpens | (Kützing) Lange-Bertalot | small | high |
| Fragilaria capucina var. vaucheriae | (Kützing) Lange-Bertalot | small | high |
| Frustulia erifuga | Lange-Bertalot & Krammer | small | motile |
| Fragilaria famelica var. famelica | (Kützing) Lange-Bertalot | small | high |
| Fragilariforma constricta | Williams & Round | small | high |
| Fragilaria gracilis | Østrup | small | high |
| Fragilaria species |  | small | high |
| Fragilaria rumpens | (Kützing) G.W.F. Carlson | small | high |
| Frustulia saxonica | Rabenhorst | small | motile |
| Fragilaria tenera | (W. Smith) Lange-Bertalot | small | high |
| Fragilaria virescens | Ralfs | small | high |
| Gomphonema acuminatum | Ehrenberg | small | high |
| Gomphonema clavatum | Ehrenberg | small | high |
| Gomphonema duplipunctatum | Lange-Bertalot & Reichardt | small | high |
| Gomphonema exilissimum s.l. |  | small | high |
| Gomphonema hebridense | Gregory | small | high |
| Gomphonema species |  | small | high |
| Gomphonema parvulum | (Kützing) Kützing | small | high |
| Gomphonema pseudobohemicum | Lange-Bertalot & Reichardt | small | high |
| Gomphosphenia stoermeri | Kociolek & Thomas | small | high |
| Gomphonema pumilum s.l. |  | small | high |
| Karayevia laterostrata | (Hustedt) Bukhtiyarova | small | low |
| Karayevia suchlandtii | (Hustedt) Bukhtiyarova | small | low |
| Karayevia oblongella | M. Aboal | small | low |
| Kobayasiella parasubtilissima | Lange-Bertalot | large | motile |
| Kobayasiella subtilissima | Lange-Bertalot | large | motile |
| Luticola acidoclinata | Lange-Bertalot | n.c. | n.c. |
| Meridion circulare var. constrictum | (Ralfs) Van Heurck | small | high |
| Meridion circulare var. circulare | (Greville) C.A. Agardh | small | high |
| Microcostatus krasskei | (Hustedt) Johansen & Sray | large | motile |
| Microcostatus maceria | Lange-Bertalot, Kusber & Metzeltin | large | motile |
| Navicula angusta | Grunow | large | motile |
| Nitzschia acidoclinata | Lange-Bertalot | small | motile |
| Naviculadicta species |  | small | motile |
| Navicula species |  | large | motile |
| Nitzschia bavarica | Hustedt | large | motile |
| Navicula cryptocephala | Kützing | large | motile |
| Navicula difficillima | Hustedt | large | motile |
| Neidium species |  | small | motile |
| Navicula festiva | Krasske | large | motile |
| Navicula heimansioides | Lange-Bertalot | large | motile |
| Nitzschia gracilis | Hantzsch | large | motile |
| Nitzschia perminuta | (Grunow) M. Peragallo | small | motile |
| Nitzschia linearis var. subtilis | (Grunow) Hustedt | large | motile |
| Navicula rhynchocephala | Kützing | large | motile |
| Navicula schmassmannii | Hustedt | large | motile |
| Navicula tenelloides | Hustedt | large | motile |
| Nupela fennica | (Hustedt) Lange-Bertalot | n.c. | motile |
| Nupela species |  | n.c. | motile |
| Naviculadicta Iconogr. 2, Taf. 28:6-9 |  | small | motile |
| Orthoseira species |  | n.c. | high |
| Psammothidium altaicum | (Poretzky) Cleve-Euler | small | low |
| Psammothidium chlidanos | Lange-Bertalot | small | low |
| Psammothidium didymum | Bukhtiyarova & Round | small | low |
| Pinnularia divergens var. divergens | W. Smith | large | motile |
| Pinnularia divergentissima var. divergentissima | (Grunow) Cleve | large | motile |
| Peronia fibula | (Brébisson & Kützing) Ross | small | high |
| Pinnularia grunowii | Krammer | large | motile |
| Psammothidium levanderi | Bukhtiyarova & Round | small | low |
| Pinnularia marchica | Ilka Schönfelder | large | motile |
| Psammothidium marginulatum | Bukhtiyarova & Round | small | low |
| Psammothidium curtissimum | (Carter) Aboal | small | low |
| Psammothidium perpusillum | (Oestrup) Lange-Bertalot | small | low |
| Pinnularia perirrorata | Krammer | small | motile |
| Pinnularia rupestris | Hantzsch | large | motile |
| Pinnularia subinterrupta | Krammer & Schroeter | large | motile |
| Pinnularia subcapitata var. subcapitata | Gregory | large | motile |
| Psammothidium scoticum | Bukhtiyarova & Round | small | low |
| Pinnularia subcapitata var. elongata | Krammer | large | motile |
| Pinnularia sinistra | Krammer | large | motile |
| Psammothidium rechtensis | (Leclercq) Lange-Bertalot | small | low |
| Pinnularia subgibba var. undulata | Krammer | large | motile |
| Planothidium lanceolatum | Lange-Bertalot | small | low |
| Planothidium peragallii | Round & Bukhtiyarova | small | low |
| Psammothidium ventralis | Bukhtiyarova & Round | small | low |
| Pinnularia viridis s.l. |  | large | motile |
| Reimeria species |  | large | low |
| Rossithidium nodosum | (A. Cleve) Aboal | small | low |
| Rossithidium petersenii | Round & L. Bukhtiyarova & Aboal | small | low |
| Rossithidium pusillum | Round & Bukhtiyarova | small | low |
| Reimeria sinuata | (Gregory) Kociolek & Stoermer | large | low |
| Staurosira construens var. exigua | (W. Smith) Kobayasi | small | high |
| Stauroforma exiguiformis | (Lange-Bertalot) Flower, Jones & Round | small | high |
| Staurosira oldenburgiana | (Hustedt) Lange-Bertalot | small | high |
| Sellaphora pupula | Mereschkowksy | large | motile |
| Surirella roba | Leclercq | large | motile |
| Staurosira pinnata var. pinnata | Ehrenberg | small | high |
| Staurosira venter | (Ehrenberg) Cleve & Moeller | small | high |
| Stenopterobia delicatissima | (Lewis) Brébisson & Van Heurck | large | motile |
| Stauroneis legumen | (Ehrenberg) Kützing | large | motile |
| Tabellaria fenestrata | (Lyngbye) Kützing | large | high |
| Tabellaria flocculosa | (Roth) Kützing | large | high |
| Tetracyclus glans | (Ehrenberg) Mills | large | n.c. |
| Ulnaria danica | (Kützing) Compère & Bukhtiyarova | n.c. | n.c. |
| Ulnaria ulna var. ulna | (Nitzsch) P. Compère | n.c. | n.c. |
